# The yeast kinome displays scale free topology with functional hub clusters

**DOI:** 10.1186/1471-2105-6-271

**Published:** 2005-11-09

**Authors:** Robin EC Lee, Lynn A Megeney

**Affiliations:** 1 Ottawa Health Research Institute, Molecular Medicine Program, Ottawa, Canada; 2 University of Ottawa, Department of Cellular and Molecular Medicine, Ottawa, Canada

## Abstract

**Background:**

The availability of interaction databases provides an opportunity for researchers to utilize immense amounts of data exclusively *in silico*. Recently there has been an emphasis on studying the global properties of biological interactions using network analysis. While this type of analysis offers a wide variety of global insights it has surprisingly not been used to examine more localized interactions based on mechanism. In as such we have particular interest in the role of key topological components in signal transduction cascades as they are vital regulators of healthy and diseased cell states.

**Results:**

We have used publicly available databases and a novel software tool termed Hubview to model the interactions of a subset of the yeast interactome, specifically protein kinases and their interaction partners. Analysis of the connectivity distribution has inferred a fat-tailed degree distribution with parameters consistent with those found in other biological networks. In addition, Hubview identified a functional clustering of a large group of kinases, distributed between three separate groupings. The complexity and average degree for each of these clusters is indicative of a specialized function (cell cycle propagation, DNA repair and pheromone response) and relative age for each cluster.

**Conclusion:**

Using connectivity analysis on a functional subset of proteins we have evidence that reinforces the scale free topology as a model for protein network evolution. We have identified the hub components of the kinase network and observed a tendency for these kinases to cluster together on a functional basis. As such, these results suggest an inherent trend to preserve scale free characteristics at a domain based modular level within large evolvable networks.

## Background

The Barabási and Albert scale free network model is a mathematical precept that describes the innate connectivity and distribution within complex networks. These scale free networks defy the traditional random graph model of Erdös and Renyi and display a connectivity distribution where the occurrence of highly interacting components of the network, defined as nodes decay as a power law, *P*(*k*) ~ *k*^-*γ *^[1-3]. In turn, growth of a scale free network is characterized by a preferential attachment scheme in which new nodes attach to older more connected nodes with a higher probability [2,4,5]. This model facilitates a rich-get-richer schema and allows for the existence of a very important class of highly connected hubs [1,6]. These hubs are largely responsible for the non-Gaussian connectivity distribution of scale free networks and are commonly orders of magnitude more connected than the average node. The existence of the hubs also provide a robust environment that is tolerant of random attack and failure but is very sensitive to hub perturbation [3,7-10].

This scale free topology has been demonstrated in a variety of man-made networks such as the World Wide Web and the actor collaboration network [1,2]. Scale free principles have also been noted in biologic systems such as the yeast protein-protein interaction dataset and the metabolic protein network [3,6]. Nevertheless, the suitability of the static scale free construct across diverse biologic systems has been challenged as a universal principle. For example, the yeast protein interaction network has been described as a date and party hub scale free network, in which these hubs are defined by variable or consistent interactions, respectively [10]. More critically, mathematical models of network growth have shown that preferential attachment may follow a random geometric topology rather than a scale free distribution [11]. Another study uses a learning algorithm to infer duplication-mutation-complementation as the central topology mechanism in the Drosophila melanogaster protein interaction network [12]. Indeed, it has been reported that the essential proteins within the larger yeast protein interaction network form an exponential connectivity distribution rather than a scale free distribution [13]. These observations raise intriguing possibilities, one of which suggests that broader scale free systems may evolve from a compilation of sub networks of different topology. Alternatively, this non-scale free structure may be an anomaly that originates from examining essential hubs versus non-essential hubs in the framework of an already established network.

Within this context, phosphorylation dependent signal transduction pathways provide an interesting venue to examine network behavior. In eukaryotic organisms, kinase directed phosphorylation is one of the most common forms of post-translational modification and as such this protein class is noted as a vital regulator of cellular function [14-16]. In addition, kinase families are well conserved across diverse phyla, suggesting that network organization may be similarly conserved. However, phosphorylation pathways are commonly studied as linear events connecting stimulus to response through a simple ladder of molecular interactions, a concept that is based largely on experimental perturbation and observation of directly connected proteins.

As such, identification of the select kinase hubs and interaction profiling should offer an insight into the functional complexities of cellular signaling in yeast and higher eukaryotes. Here, we examined the subset of the *S. cerevisiae *interaction data, which include protein kinases and their direct protein interactions. In all cases, analysis was performed on filtered datasets available in public databases to identify likely hub kinases and their interactivity. We confirmed scale free behaviour of this dataset using connectivity analysis and observed parameters as applied to a novel computer program/visualization tool we termed Hubview [17]. Interactions between the 19 most connected kinases, which we identified as super-hubs, were mapped along with less connected hub kinases. From this map we were able to discern three distinct clusters of kinase proteins, with each cluster retaining a common biologic function, i.e. cell cycle control, DNA repair/recombination and the pheromone/mating response. Together these observations suggest that scale free topology of the yeast kinome co-evolved with the emergence of distinct biologic domains.

## Results and discussion

**Figure 1:**
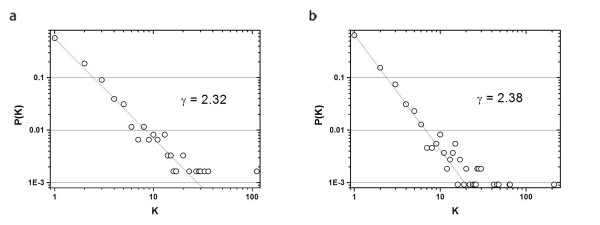
Degree distributions giving the probability that a given protein will interact with exactly k other proteins for: **a**. the core KPI with γ_core _= 2.32 **b**. the complete KPI with γ_complete_= 2.38. In both cases γ determined by maximum likeliness estimation (MLE) and goodness of fit determined by Kolomogorov-Smirnov (KS) test. The self organization of scale free topology is normally associated with much larger datasets yet we still find the scale free characteristics.

To study the topological properties of kinase mediated phosphorylation it was necessary to isolate the signaling component of the S. Cerevisiae proteome which we refer to as the *Kinase-Partner Interaction *set (KPI). The KPI node set was assembled from the concatenation of kinases from the database of interacting proteins (DIP) kinase search, the yeast kinases identified by Hunter and Plowman [18] and their non-kinase interaction partners. The interactions of the KPI nodes were considered bidirectional, as no directionality can be consistently inferred in most experimental conditions, and consisted of kinase-kinase and kinase-non-kinase interactions only (i.e. any potential interactions between non-kinases have been filtered). The core and complete KPI consisted of 607 nodes with 834 interactions and 1085 nodes and 1481 interactions respectively. Analysis using maximum likelihood estimation (MLE) of the degree distributions (Figure 1) resulted in derived γ values of γ = 2.32 in the core KPI and γ = 2.38 in the complete KPI which is in the biologically robust range of 2 < γ < 3 [19]. The Kolmogorov-Smirnov test for Power Law Distribution [20] of both MLEs (Core: N = 500, K = 0.021; Complete: N = 1000, K = 0.015) support the hypothesis that the KPI networks are indeed both power-law distributions and hence scale free in topology. The γ-values found for the KPIs are very consistent with reports from complete protein interaction data analysis and the deterministic scale free model [21,22] which confirms that the selection criteria for the KPI is not biased to any connectivity class. A study of metabolic networks has shown that the largest most connected part of a network (in the case of metabolic networks the largest component is less than 33% the size of the full network) tends to dominate the parameters found through topological analysis [23]. Here the degree distribution is challenged as a global property and treated as a local property of the network. It is worth noting that while the KPI does contain a limited number of segregated modules, the size of the largest component accounts for roughly 95% of the network and the degree distribution does represent a global property of the network.

**Figure 2:**
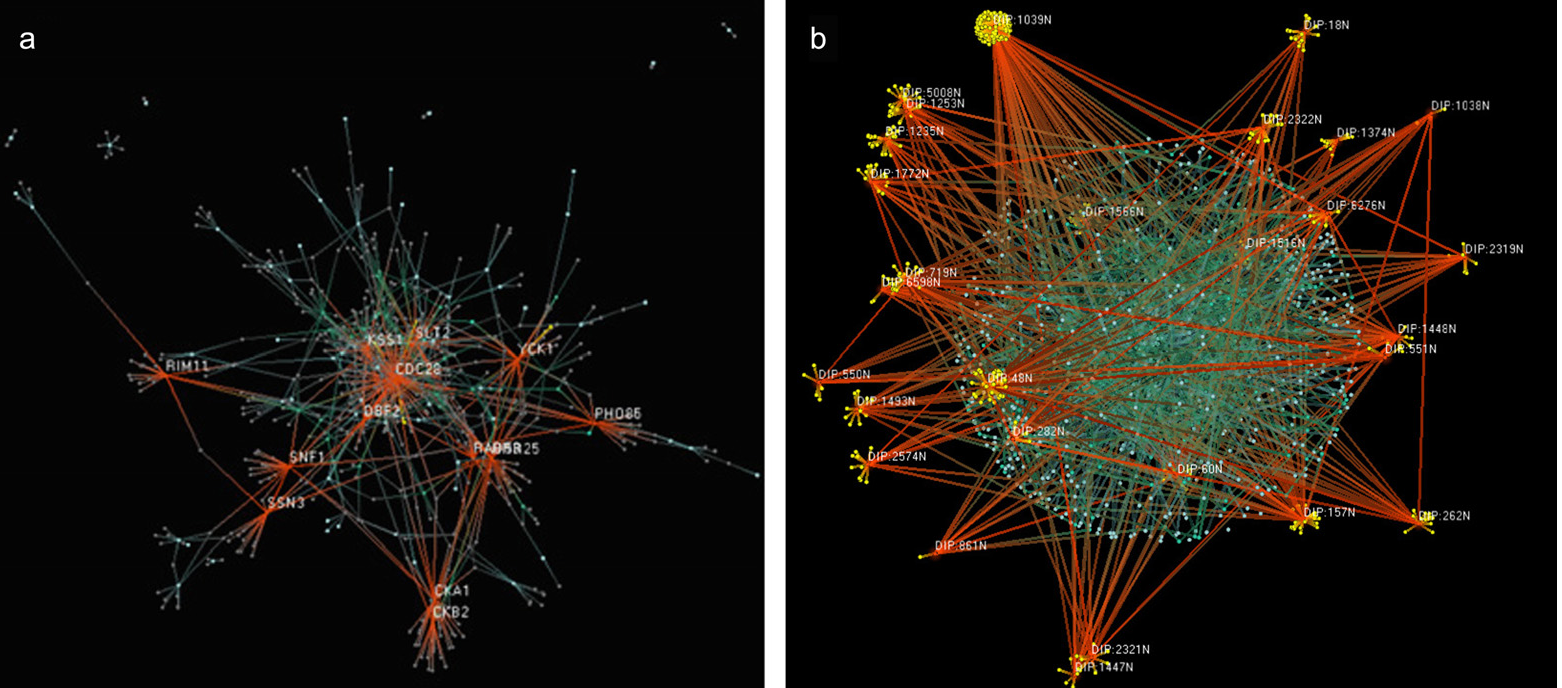
**a**. Hubview Fruchterman-Rheingold visualization of Core KPI (607 nodes, 834 interactions) **b**. Hub-Star-Satellite output of Hubview of complete KPI (1085 nodes with 1481 interactions) with hub degree cut-off of 15 yields 28 hubs.

The KPI interaction data was analyzed by our visualization tool, Hubview. The hub-star-satellite view separates nodes with degrees higher than a user defined cut-off and their substrates of unary degree, groups the rest of the nodes within a sphere, and places the hub-stars around the sphere as satellites. The core and complete KPI were viewed with a cut-off of 10 and 15 respectively (Figure 2) and in both cases resulted in 28 satellites responsible for about 69% and 71% of the interactions respectively. The average node degree for both the core and complete KPI was found to be <k> ≅ 1.3.

The putative hubs identified by Hubview were compiled into a list of 33 distinct nodes and ranked by average degree where the degree found in the core KPI was given twice the weight (Table 1). Defining the actual cut-off degree for a hub is a subjective task, here we defined 13 (10*<k>) as the cut-off for high confidence in super-hub status. This cut-off retains 19 proteins as high confidence hubs that still maintain ~64% of KPI interactions which suggests that less understood signaling systems in higher eukaryotes may be studied with higher efficiency by identifying likely hub kinases (using expression and activity profiling) and mapping the complete set of their immediate interaction partners.

**Table 1:** Summary of hub kinases as identified by Hubview: Weighted mean calculated by giving double weight to degrees listed in the core KPI dataset. Hubs with knockout lethal phenotype listed as identified by Giaver et al [35].

**Name**	**DIP Node Number**	**Knockout Lethal**	**Weighted Degree**	**Confidence as a super-hub**
CDC28	DIP:1039N	yes	150 ± 40	High
CKA1	DIP:48N	no	50 ± 10	High
HRR25	DIP:157N	yes	40 ± 10	High
SLT2	DIP:1448N	no	37 ± 5	High
YCK1	DIP:719N	no	34 ± 7	High
KSS1	DIP:60N	no	33 ± 5	High
SNF1	DIP:18N	no	24 ± 1	High
PHO85	DIP:1493N	no	22 ± 2	High
RAD53	DIP:2322N	yes	22 ± 2	High
CKB2	DIP:262N	no	19 ± 5	High
CDC7	DIP:1235N	yes	19 ± 6	High
RIM11	DIP:1566N	no	19 ± 3	High
SSN3	DIP:2574N	no	17	High
DBF2	DIP:2319N	no	17 ± 2	High
DUN1	DIP:1772N	no	16 ± 6	Uncertain
MKK2	DIP:1447N	no	16 ± 2	High
CDC5	DIP:2321N	yes	14 ± 1	High
STE11	DIP:861N	no	14 ± 1	High
PKC1	DIP:1516N	yes	14 ± 1	High
TPK3	DIP:550N	no	14 ± 1	High
CKA2	DIP:1038N	no	13 ± 5	Uncertain
SPS1	DIP:6598N	no	13 ± 2	Uncertain
CLA4	DIP:2276N	no	13	Uncertain
YAK1	DIP:1374N	no	12 ± 4	Uncertain
STE20	DIP:712N	no	12 ± 1	Uncertain
FUS3	DIP:714N	no	12 ± 1	Uncertain
CHK1	DIP:1253N	no	12 ± 2	Uncertain
KIN2	DIP:6276N	no	12 ± 2	Uncertain
BUD32	DIP:5008N	no	11 ± 9	Uncertain
SWE1	DIP:2410N	no	11	Low
CKB1	DIP:282N	no	11 ± 7	Uncertain
GIN4	DIP:2260N	no	11 ± 1	Low
BCY1	DIP:551N	no	10 ± 3	Low

The 124 members of the protein kinase superfamily list [18] were cross referenced with the list of essential yeast proteins [24] to identify the yeast kinases with known knockout lethal phenotypes. Of the 124 kinases only 16 were deemed to be lethal deleterious mutants yielding a 13% chance of lethality in an instance of random single kinase deletion. In contrast, 6 of the 19 hubs named as *high confidence *in table 1 are listed as essential resulting in a 32% chance of lethality attributed to random deletion of one of the 19 high confidence hubs. This marked increase in lethality associated with directed hub attack is consistent with existing studies of scale free networks [3] and indicates a likely tendency for hub kinases to be preserved in an evolutionary perspective.

**Figure 3:**
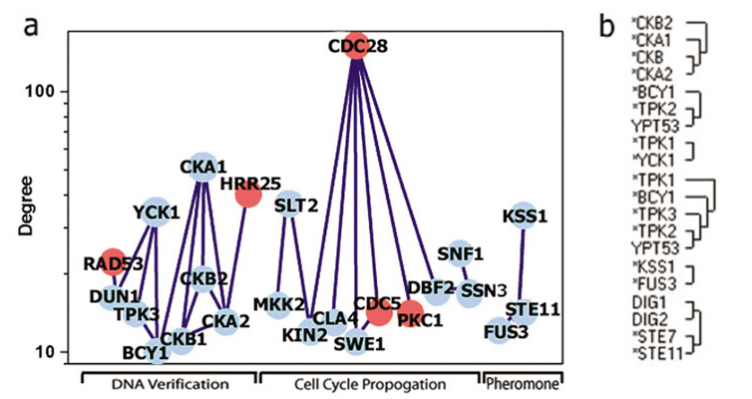
**a**. Interplay between 24 of the hubs identified by HubView constituting the signaling backbone. Red hubs are deemed as lethal knockout phenotypes as described in the systematic deletion project [35]. The nodes are separated by degree along the ordinate to illustrate a possible relationship between the degree of a hub and its phylogenetic age while the arrangement along the abscissa is purely aesthetic. The proposed function of the 3 clusters from left to right are: DNA damage/repair, cell cycle propagation and pheromone response. **b**. The dendrogram to the right confirms functional interactions for a number of the backbone hubs using redundancy clustering of the entire KPI as described by Samanta & Liang [28]. Only clusters containing a hub kinase are depicted in the dendrogram.

24 of the 33 hubs listed in table 1 were found to interact with one another. The interplay between these 24 connected hubs forms a kinase signaling backbone (figure 3a) through which 3 distinct groups of interacting hubs (forthwith these interacting hubs are referred to as hub clusters) can be identified. Presumably, the hub clusters would provide vital functions as whole as in most cases the constituent hubs are not directly essential themselves. The structure of this backbone may offer some insight in identifying synthetic lethality strategies, i.e. CKA1 and CKA2 knockouts are both viable but double deletion mutant has a lethal phenotype [25]. Backbone hubs have been ordered by degree to illustrate a possible correlation between degree and phylogenetic age where, by direct consequence of the growth and preferential attachment conditions in scale free systems, more connected hubs are likely to be older than less connected hubs [26]. A cross genome study of four organisms from different regions of the phylogenetic tree has been used to identify connectivity and emergence time of yeast proteins [5]. The results of this study support the preferential attachment and growth criteria as outlined by the scale free theory. Older proteins appear to be more connected than younger proteins. Another explanation of the degree arrangement is that the average size or degree of a cluster is associated with the evolutionary age of the clusters functional class [27]. This perspective is based on a similar study using a more rigorous phylogenetic profiling technique. The results suggest a modified form of scale free preferential attachment whereby proteins bind preferentially within their own functional class and not globally or promiscuously. By this model a younger protein may be more connected than an older one simply because it is part of an older and more connected functional grouping which emerged during an earlier phylogenetic period. Here the average connectivity of the functional group is proportional to the age of that group i.e. older eukaryotic proteins are shown to be more connected than yeast specific proteins. This perspective is very plausible as it suggests that proteins of similar function will interact within the same pool.

In response to the latter interpretation we examined the basic purpose of the individual hubs and observed a common functional theme concomitant with each cluster. The largest cluster, containing cdc28, is functionally associated with cell cycle propagation through the various phases. The second cluster, with CKA1 as a peak, is generally associated with kinase proteins that manipulate response to DNA damage and the final KSS/MAPK cluster is involved with the regulation of the pheromone response. These results seem to offer a reasonable order to the emergence of specialized functions central to all eukaryotes i.e., the cell division cycle predates the DNA verification mechanisms, which in turn predates the youngest reproductive module, the mating response.

The entire core and complete kinomes were clustered using the probabilistic method described by Samanta and Liang [28]. This method identifies functional relationships between proteins through redundancy of interaction partners. A number of the associations in the backbone clusters were confirmed using this algorithm (figure 3b). Interestingly the proteins in the cell cycle propagation cluster did not appear as functionally redundant in the clustering. Presumably the three clusters converge downstream to some extent but at the hub level this indicates that these components offer highly specialized non redundant services to the cell cycle cluster likely due to the ancient nature of their function. This method can also be used to identify likely synthetic lethality as many viable knockouts are rescued through redundant interactions. The full results of the clustering is available as supporting information (see additional file 1) or can be generated using Hubview.

**Figure 4:**
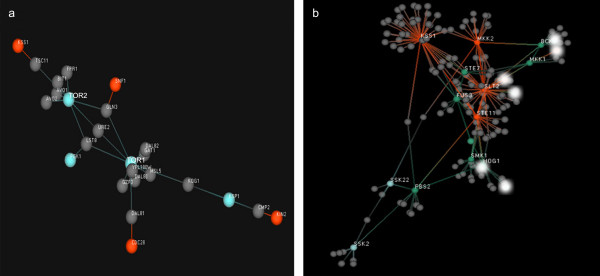
Cascade crawler output of Hubview. Gray spheres represent non-kinases while all other colored spheres represent kinases of varying degree. a) Depiction of closed loop TOR signaling: neither TOR protein is directly connected to another kinase indicating highly specified reaction. b) Depiction of open loop MAPK signaling: white spheres denote non-MAPK kinases that interact within and outside of the MAPK clusters representing possible regulatory and cross-talk channels associated with more complicated cellular behaviour.

In addition to the scale free topology, modeling of the yeast kinome using the Hubview cascade crawler function revealed other notable characteristics. Specifically, individual clusters containing hub kinases also include kinases that interact both inside and outside the scope of the immediate functional cluster. This characteristic was generally not observed with non-hub clusters. For example, the cluster of kinases involved in the MAPK cascade (a functional cluster with hub kinases) retain interactions with a number of non-MAPK kinases i.e. single points that interact both within and outside of the MAPK class. This is a feature we refer to as an open loop signal (Figure 4). Identifying open points within a cluster provides the user with probable targets for regulation of that functional cluster or even likely paths for signaling crosstalk. Open loop kinase cascades appear to reflect robust cellular responses that require multiple alterations and as such would require direct communication and signal propagation between numerous key regulatory factors/kinases themselves. However, non hub kinase clusters such as the TOR kinase cascade do not retain direct interactions between unclustered kinases and as such conform to a closed loop structure (Figure 4). Closed loop structures are likely to be kinase directed cascades that perform a very discrete cellular function in response to a limited or very specific initiating event.

The network of essential yeast proteins has been compiled and identified as an exponential distribution [13]. This distribution is normally associated with more stochastic evolutionary mechanisms. It has been argued that this network may represent an ancestral core about which the rest of the yeast interactome has formed [13]. The existence of an exponential core does not directly contradict the scale free topology observed in the protein interaction network but may simply exist as a scaffold for scale free mechanisms to adhere to. This possibility is interesting as it may also suggest that different parts of the interactome may have evolved by different evolutionary pressures causing unequal distribution of topological properties within the same interactome.

A recent investigation concerning the effects of sampling on topology adds a small shard of doubt to studies of protein network topology. In this study the effects of various large scale experiments were simulated by first generating different networks of known topology and then sampling interactions in a scale mimicking yeast two hybrid and co-affinity purification [29]. They found that under some conditions that non-scale free topologies (i.e. Erdös and Renyi network with <K> = 10), when sampled, can generate sub-networks with scale free properties. Here the kinome benefits from the fact that it is a widely studied mechanistic class and many of the interactions, especially in the core kinome, have been identified in smaller scale experiments and not exclusively large scale experiments. This suggests that the much smaller kinome network may not suffer as much as networks derived solely from large scale experiments. The results of this study certainly insist on the caveat that the results of our KPI network cannot be extrapolated to the complete yeast protein interaction network with any amount of confidence.

## Conclusion

Our analysis suggests that the yeast kinome is an evolved scale free system. Moreover, these observations suggest the intriguing possibility that the scale free topology of the global protein-protein interaction network or any larger biologic network may be the composite of smaller evolving topologies (such as the kinome), all of which are subject to their own selective pressures.

## Methods

### Interaction database

Both the core and complete yeast interaction data of the manually curated DIP [30] were used as interaction data sets. The complete dataset consists largely of high throughput interaction data [19,31-33]. The core DIP dataset consists of interactions found in small scale experiments, two or more independent larger scale experiments and, when paralogous interaction data exist, the Paralogous Verification Method PVM [31,32]. The core dataset is believed to correctly identify the core of interacting proteins in yeast and provides a *minimal interaction *view of the yeast interactome. For our purposes the complete yeast interaction set is viewed as a hypothetical *maximal interaction *set. The many false positives, negatives and unlikely biologic interactions [19] available in the complete dataset are still valuable as they may be representative of interactions in a diseased state based on possible spatial and temporal protein delocalization. The DIP is available online at .

### Interaction filter

Both datasets were filtered to include only kinases and direct interaction partners with kinases as found in the DIP node search in conjunction with kinases listed in the protein kinase superfamily found by Hunter and Plowman [18]. The resulting *Kinase-Partner Interaction *dataset (KPI) consisted of 607 nodes with 834 interactions in the case of the core dataset and 1085 nodes with 1481 interactions in the case of the complete dataset.

### Hubview description

We developed a program called *Hubview *to help us analyze the KPI network and visualize the hubs and hub interactions found in the datasets. The degree distribution of the loaded network can be obtained by pressing the *probability distribution *button. The main program and OpenGL network interface utilize an undirected binary adjacency matrix which is then interpreted in real-time 3D. Yeast specific information such as the naming convention (DIP number, ORF and common name) and protein type (kinase or non-kinase) is hard coded into Hubview minimizing the amount of data required to generate an interaction network. The 3D representation is geared towards identifying nodes with degrees higher than a user-defined cut-off and displaying them in either a hub-star-satellite view whereby hub degree and inter-hub interactions are plainly visible or a Fruchterman-Rheingold (FR) force-directed placement arrangement [34] which offers a less tangled, more visually appealing interpretation.

Briefly, the FR algorithm causes the system to untangle itself through iterative simulation of mechanical and electrostatic forces. A connection between a pair of nodes is treated as though a spring were connecting those nodes creating attractive forces between all connected pairs. Repulsive electrostatic forces are also generated by considering each node as a negative point charge. The nodes in the analogous system move in 3 dimensional space according to the attractive and repulsive forces. The final arrangement is displayed once the system has evolved through a set number of iterations resulting in an intelligible and appealing graph.

The hub-star-satellite view is generated by placing all nodes randomly within a sphere of radius r_i_. All nodes with connectivity higher than the user defined cutoff are identified as hubs and projected outside of the sphere to a radial position, r_f_, outside the confines of the initial sphere (r_f _> r_i_). Any substrates of the new hub with unary degree are also moved to positions spherically centered near the newly placed hub generating a hub-star-satellite. The algorithm ends once all hubs are processed similarly. The advantage of this view type is that it allows interactions between hubs to be quickly and easily identified as all visually interfering substrates remain pooled within the initial sphere.

Another useful visualization method included in Hubview is the cascade crawler function. This view type is geared towards depiction of smaller cascades (the immediate and remote neighbors of a chosen protein) within the complete network. The cascade crawler function is controlled by a point and click interface whereby the user can define a specific protein(s) as a starting point and display all of its substrates by clicking on it. Clicking subsequent nodes will display their interaction partners in turn. Using this function along with the FR algorithm one can develop appealing visual interpretations of specific cascades and interactions (figure 4).

Hubview also utilizes the clustering method proposed by Samanta & Liang [28]. The main suggestion of this algorithm is that if two proteins in a network share a significantly larger number of common interaction partners than what is expected from a similar random network then the pair of proteins likely share a close functional relationship. This process assigns a P value between every pair of proteins in the network representing the probability that an association between proteins is random i.e. a higher P score means that the pair is not functionally associated. The algorithm then merges the pair sharing the lowest P value into a cluster and recalculates P values for all possible pairs again treating the newly formed cluster as though it were a single protein. This process repeats until all P values are higher than a user defined cutoff. Once a network is loaded one can access this method by clicking the cluster button. Here a cutoff value can be defined which represents the probability that a particular association is random and a dendrogram is produced (which can be saved as a .BMP file), Samanta & Liang reported successful clustering of a large portion of the yeast interactome (N = 4692) using a cutoff value of up to 2 × 10^-4 ^[28] indicating that this cutoff can be considered sharp and biologically relevant in our much smaller KPI networks (N_core _= 607 and N_complete _= 1085).

### Topology analysis

To counter the distortion associated with log-log data transformation the γ-value associated with the degree distribution of the *KPI *was analyzed using maximum likelihood estimation of the zeta function (MLE) and goodness of fit confirmed by the Kolmogorov-Smirnov test for power law distributions [20]. Briefly, the γ parameter associated with the pure power law,

P(k)=k−γζ(γ)     [1]
 MathType@MTEF@5@5@+=feaafiart1ev1aaatCvAUfKttLearuWrP9MDH5MBPbIqV92AaeXatLxBI9gBaebbnrfifHhDYfgasaacH8akY=wiFfYdH8Gipec8Eeeu0xXdbba9frFj0=OqFfea0dXdd9vqai=hGuQ8kuc9pgc9s8qqaq=dirpe0xb9q8qiLsFr0=vr0=vr0dc8meaabaqaciGacaGaaeqabaqabeGadaaakeaacqWGqbaucqGGOaakcqWGRbWAcqGGPaqkcqGH9aqpdaWcaaqaaiabdUgaRnaaCaaaleqabaGaeyOeI0cccaGae83SdCgaaaGcbaGae8NTdONaeiikaGIae83SdCMaeiykaKcaaiaaxMaacaWLjaGaei4waSLaeGymaeJaeiyxa0faaa@3FED@

is best approximated by the solution of:

∂ζ(γ)∂γζ(γ)=−∑i=1NLog(ki)N     [2]
 MathType@MTEF@5@5@+=feaafiart1ev1aaatCvAUfKttLearuWrP9MDH5MBPbIqV92AaeXatLxBI9gBaebbnrfifHhDYfgasaacH8akY=wiFfYdH8Gipec8Eeeu0xXdbba9frFj0=OqFfea0dXdd9vqai=hGuQ8kuc9pgc9s8qqaq=dirpe0xb9q8qiLsFr0=vr0=vr0dc8meaabaqaciGacaGaaeqabaqabeGadaaakeaadaWcaaqaamaaliaabaGaeyOaIylccaGae8NTdONaeiikaGIae83SdCMaeiykaKcabaGaeyOaIyRae83SdCgaaaqaaiab=z7a6jabcIcaOiab=n7aNjabcMcaPaaacqGH9aqpcqGHsisldaWcaaqaamaaqahabaGaemitaWKaem4Ba8Maem4zaCMaeiikaGIaem4AaS2aaSbaaSqaaiabdMgaPbqabaaabaGaemyAaKMaeyypa0JaeGymaedabaGaemOta4eaniabggHiLdGccqGGPaqkaeaacqWGobGtaaGaaCzcaiaaxMaacqGGBbWwcqaIYaGmcqGGDbqxaaa@526C@

Where:

- ζ(γ) is the Riemann Zeta function

∑k=1∞k(−γ)     [3]
 MathType@MTEF@5@5@+=feaafiart1ev1aaatCvAUfKttLearuWrP9MDH5MBPbIqV92AaeXatLxBI9gBaebbnrfifHhDYfgasaacH8akY=wiFfYdH8Gipec8Eeeu0xXdbba9frFj0=OqFfea0dXdd9vqai=hGuQ8kuc9pgc9s8qqaq=dirpe0xb9q8qiLsFr0=vr0=vr0dc8meaabaqaciGacaGaaeqabaqabeGadaaakeaadaaeWbqaaiabdUgaRnaaCaaaleqabaGaeiikaGIaeyOeI0cccaGae83SdCMaeiykaKcaaaqaaiabdUgaRjabg2da9iabigdaXaqaaiabg6HiLcqdcqGHris5aOGaaCzcaiaaxMaacqGGBbWwcqaIZaWmcqGGDbqxaaa@3E44@

- k_i _is the i^th ^non-zero observed degree of the P(k) vs. k distribution.

- γ is the power law exponent [20]

### Protein essentiality

Phenotypic profiles of gene-deletion mutants (nearly 96% of known ORFs) have been systematically constructed and analyzed by a PCR-based gene deletion strategy [35]. A list of essential ORFs has been generated [24] and can be used to predict a lethal protein knockout or disruption phenotype.

## Authors' contributions

RECL participated in the projects design and coordination, carried out programming, informatics, and drafted the manuscript. LAM conceived the study, participated in its design and coordination and drafted the manuscript.

## Supplementary Material

Additional File 1 The software Hubview has been successfully tested and used on a number of recent generation PCs with the Windows XP operating system. Suggested systems should have more than 256 Megs of ram and an OpenGL compliant video card with onboard ram. The software requires the windows operating system. Installation: Simply unzip all files into the same folder. Supplementary Material.doc: Microsoft Word Document, Complete results of redundancy clustering. Hubview.zip: Winzip archive, Reviewer copy of Hubview program used to develop data for this manuscript. Click here for file
