# Supplementary material for: The yeast kinome displays scale free topology with functional hub clusters
Source: BMC Bioinformatics. 2005 Nov 9;6:271. doi: 10.1186/1471-2105-6-271 (PMC1310538; doi:10.1186/1471-2105-6-271)
Supplement: Additional File 1 — The software Hubview has been successfully tested and used on a number of recent generation PCs with the Windows XP operating system. Suggested systems should have more than 256 Megs of ram and an OpenGL compliant video card with onboard ram. The software requires the windows operating system. Installation: Simply unzip all files into the same folder. Supplementary Material.doc: Microsoft Word Document, Complete results of redundancy clustering. Hubview.zip: Winzip archive, Reviewer copy of Hubview program used to develop data for this manuscript. [file 1471-2105-6-271-S1.doc]

**Supplementary Material**

**Clustering of the Core & Complete KPI by Redundancy**

The clustering algorithm uses a cutoff value which represents the probability that a particular association is random. Samanta & Liang reported successful clustering of a large portion of the yeast interactome (N_Samanta-Liang_ = 4692) using a cutoff value of up to 2 × 10^-4^ indicating that this cutoff can be considered sharp and biologically relevant in our much smaller KPI networks (N_core_ = 607 and N_complete_ = 1085).


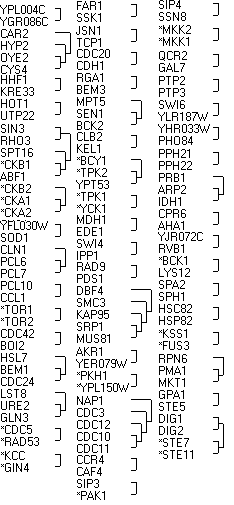


49 clusters using the core KPI with a cutoff of 2×10^-4^. Prefix of ‘*’ indicates a kinase.

**
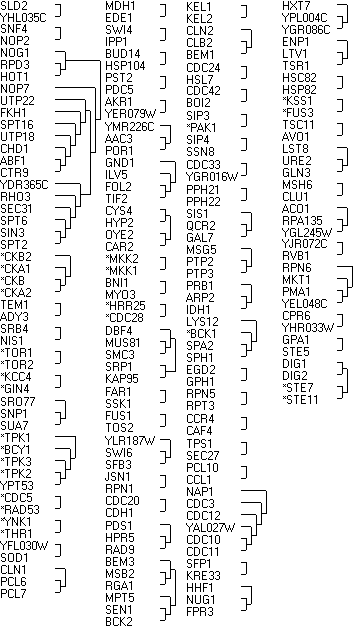
**

64 clusters using complete kinome with cutoff of 2×10^-4^. Prefix of ‘*’ indicates a kinase.
